# A neural mechanism for affective well-being: Subgenual cingulate cortex mediates real-life effects of nonexercise activity on energy

**DOI:** 10.1126/sciadv.aaz8934

**Published:** 2020-11-06

**Authors:** Markus Reichert, Urs Braun, Gabriela Gan, Iris Reinhard, Marco Giurgiu, Ren Ma, Zhenxiang Zang, Oliver Hennig, Elena D. Koch, Lena Wieland, Janina Schweiger, Dragos Inta, Andreas Hoell, Ceren Akdeniz, Alexander Zipf, Ulrich W. Ebner-Priemer, Heike Tost, Andreas Meyer-Lindenberg

**Affiliations:** 1 Department of Psychiatry and Psychotherapy, Central Institute of Mental Health, Medical Faculty Mannheim, Heidelberg University, Mannheim, Baden-Wuerttemberg, Germany.; 2 Mental mHealth Lab, Chair of Applied Psychology, Institute of Sports and Sports Science, Karlsruhe Institute of Technology (KIT), Baden-Wuerttemberg, Germany.; 3 Department of Bioengineering, University of Pennsylvania, Philadelphia, PA, USA.; 4 Division of Biostatistics, Central Institute of Mental Health, Medical Faculty Mannheim, Heidelberg University, Mannheim, Baden-Wuerttemberg, Germany.; 5 Institute of Geography, GIScience Research Group, Heidelberg University, Heidelberg, Baden-Wuerttemberg, Germany.

## Abstract

Physical activity substantially improves well-being and mental health, but the underlying brain processes remain unclear. Most research concerns exercise, although the majority of everyday human behaviors, such as walking or stair climbing, are nonexercise activities. Combining neuroimaging with ecological assessment of activity and GPS-triggered smartphone diaries, we show a specific association of nonexercise activity with energy in two independent samples mediated by the subgenual part of the anterior cingulate cortex (sgACC), a key emotion regulatory site. Furthermore, energy predicted a range of mental health metrics. sgACC volume moderated humans’ emotional gain from nonexercise activity in real life: Individuals with low sgACC volume, a risk factor for depression, felt less energized when inactive but benefited more from periods of high nonexercise activity. This suggests an everyday life mechanism affecting affective well-being in the general population and, if substantiated in patient samples, a risk and resilience process for mood disorders.

## Teaser

A neural mechanism underlying the real-life effects of nonexercise activity on well-being promises novel intervention targets.

## INTRODUCTION

Being physically active comes with not only several physiological and somatic but also psychological benefits. For example, physical activity increases affective well-being in the general population (*1*, *2*) and even reduces the incidence rates of several psychiatric conditions (*3*). To do justice to the behavioral repertoire, one needs to distinguish between subtypes of physical activity that differ with regard to their physiological and psychological processes, e.g., the (social) context, motive, structure, duration, and energy expenditure (*4*). Clearly, exercise activities such as playing soccer with friends every Monday and Thursday evening differ from nonexercise activities such as spontaneously fetching papers from the basement at work. Accordingly, exercise activities are defined as structured physical activities with high demands of energy expenditure across prolonged time periods (*4*), whereas nonexercise activities comprise all other daily physical activities (e.g., climbing stairs, gardening, and catching the train), which are “often processed automatically and habitually or performed spontaneously” (*4*). However, epidemiological studies have ignored this distinction and often considered physical activity as a global construct comprising all human physical activities (*1*, *2*). Similarly, clinical trials focused on the impact of physical exercise on mental health outcomes (*5*, *6*), ignoring the potential effects of nonexercise activities on affective well-being and mental health.

Thus, although influences of physical exercise on affective well-being and mental health have been known for centuries and have been a research topic for decades, the specific impact of nonexercise activities on affective well-being and mental health benefits is only recently being studied. This is unexpected, given that nonexercise activities constitute the predominant part of humans’ everyday life physical activity. As has been argued by public health research, nonexercise activity may thus be an intervention target that can be modified more easily than time-consuming and exhausting physical exercise sessions. Part of this neglect may lie in the difficulty of measuring nonexercise activity in real life. However, this obstacle is increasingly overcome as digital progress allows objective assessment of nonexercise activity and its dynamic association with affective well-being within persons over time with ambulatory assessment. This uses accelerometry for nonexercise activity measurement and electronic diaries for the repeated real-time assessments of psychological states in everyday life (*4*, *7*), avoiding limitations of traditional methods such as retrospective distortions and limited ecological validity of laboratory findings (*8*).

Ambulatory assessment studies on physical activity and affective well-being and mental health within the past two decades mainly tackled the real-life relationship between physical activity and mood, showing that both constructs are associated within humans across time (*9*). The most prominent evidence suggests that momentary bouts of increased physical activity coincide with enhanced feelings of energy in everyday life (*4*, *10*, *11*). From a clinical perspective, this is especially important for affective disorders, where both reduced physical activity and diminished feelings of energy are closely related and constitute central diagnostic criteria (*12*). Moreover, patients diagnosed with major depression show reduced physical activity in comparison to healthy controls (*13*, *14*), and treatment response in this population has been related to increased physical activity (*13*, *14*). A recent ambulatory assessment study (*15*) identified interactions between physical activity and perceived energy as a previously unidentified intervention target for mental health. In particular, among healthy participants as well as patients with major depression and bipolar disorder, cross-domain reactivity over 24-hour periods was especially found between physical activity and feelings of energy as indicated by bidirectional associations of physical activity with energy levels. In summary, the association between physical activity and energetic arousal in everyday life appears as a highly promising target not only for the improvement of affective well-being but also for mental health prevention and intervention. Our own prior ambulatory assessment studies provide indications that momentary positive effects of physical activity on energetic arousal in real life are specifically driven by nonexercise activities but not by exercise in the general population.

However, a useful understanding of the processes linking nonexercise activities and affective well-being would benefit from a knowledge of brain circuits underlying these psychological real-life mechanisms. This topic, however, is understudied. While physical activity, in general, has been demonstrated to affect total brain gray matter volume (*16*) and, particularly, the hippocampal formation, subtypes of physical activity affect the brain differently (*17*).

In a discovery study, we first aimed to substantiate prior indications of a specific positive within-subject association between momentary nonexercise activity and energetic arousal in everyday life (*4*, *9*, *18*). Therefore, we equipped 67 participants with hip accelerometers measuring nonexercise activities and GPS-triggered electronic diaries querying for energetic arousal repeatedly in real time across 1 week in their everyday life. We expected a positive within-subject effect of momentary nonexercise activity on subsequent feelings of energy and, in contrast, no positive within-subject association of physical exercise with energy.

In a second study, to investigate the neurobiological underpinnings of nonexercise activity and its dynamic within-subject association with energetic arousal in real life, we subjected another 83 participants to the same ambulatory assessment procedure, again for 7 days, and we additionally measured brain gray matter volume thereafter. In this replication study, we used voxel-based morphometry to assess associations between nonexercise activity and brain gray matter volume. Building on the evidence for a link between nonexercise activities and affective well-being, we specifically examined associations of nonexercise with neural circuits involved in emotion regulation (*19*, *20*)or mood disorders (*21*) that have been shown also to be associated with physical activity (*16*, *17*): the anterior cingulate cortex (ACC), prefrontal and premotor areas, the amygdala, the thalamus, and the hippocampus. We expected that the participants’ individual total amount of nonexercise activity across the study week relates to gray matter volume differences in these brain areas (hypothesis I). Moreover, we hypothesized that the individual gray matter volume of brain areas linked to nonexercise activity (hypothesis I) would moderate differences in within-subject associations of momentary nonexercise activity with energetic arousal between individuals (hypothesis II). Last, to explore the relevance for affective well-being and mental health, we tested for associations of the within-subject target energy with established well-being and mental health metrics, expecting positive associations with markers of resilience and well-being indicators (hypothesis III). For details on the research plan, see fig. S1.

## RESULTS

### Discovery study to substantiate prior indications of a specific association of nonexercise activity and energetic arousal

To test whether the association of physical activity and energetic arousal is indeed driven by nonexercise activities, we acquired intensive longitudinal data in a discovery study (*n* = 67; for details on sample characteristics, see table S3). Participants from the discovery study showed engagement in nonexercise activities, which is in line with other healthy cohorts (*22*); on average, they performed 73.55 min of physical exercises per week (with 43 of 67 participants not engaging in any physical exercise at all), and the feelings of energy as quantified by repeated real-time e-diary ratings across the study week were in line with other healthy samples (*10*, *11*). We applied a multilevel model analysis, nested the energetic arousal assessments within participants, and incorporated nonexercise activities within the 60-min segments before each e-diary prompt (parameterized as Movement Acceleration Intensity measured via the accelerometers; for details, see Methods), as well as the momentary cumulative physical exercise duration at the respective day as predictors of interest. Energetic arousal, as reported on a validated two-item short scale (range, 0 to 100) on smartphone diaries, was entered as the dependent variable (see Methods). As expected, we found a significant and positive within-subject association of nonexercise activity on subsequent feelings of energy (*P* < 0.001; see table S4), but no within-subject influence of physical exercise on energetic arousal (*P* = 0.212; see table S4), showing that momentary positive effects of physical activity on feelings of energy in everyday life are indeed driven by nonexercise activities. Control analyses showed that these results are robust against the definition of physical activities (see section S1 and tables S1 and S2).

### Replication study

In the replication study, we acquired structural magnetic resonance imaging data in addition to the ambulatory assessment data from 83 participants to investigate associations between nonexercise activity with brain gray matter volume in brain circuits implicated in affective well-being, mood disorders, and physical activity. Thereafter, we investigated whether individual gray matter volume of brain areas showing associations with nonexercise activity moderate differences in within-subject associations between nonexercise activity and energetic arousal between individuals. Participants’ average levels of nonexercise activity across the study week (see Table 1) were in line with activity data from other healthy cohorts (*22*), suggesting that participants showed representative engagement in nonexercise activities. On average, participants engaged in 138.53 min of physical exercise per week, with 33 of 83 participants not engaging in any physical exercise at all (see Table 1). Feelings of energy across the study week (see Table 1) were in line with other healthy samples (*4*, *10*, *11*).

**Table 1:** Participant characteristics and descriptives of the replication study (*n* = 83). BMI, body mass index; WBI, well-being index; NEO-FFI-30, NEO Five-Factor Inventory (short version).

	**Min**	**Max**	**Mean**	**SD**
Age (years)	18	28	23.30	2.60
BMI (kg/m^2^)	17.17	32.66	22.98	2.93
Socioeconomic status	5.5	20.4	14.21	3.56
World Health Organization WBI	2	23	15.51	4.34
Neuroticism (NEO-FFI-30-N)	0	3.83	1.21	0.76
Nonexercise activity (milli-g per participant per week)	16.37	60.71	36.08	10.16
Energetic arousal (mean per participant per week)	31.89	78.13	59.27	10.53
Exercise activity (min per participant per week)	0	1016	138.53	191.03
E-diary compliance [answers/ queries in (%)]	40.22	100	80.87	16.33

#### Nonexercise activity and sgACC gray matter volume

**Fig. 1:**
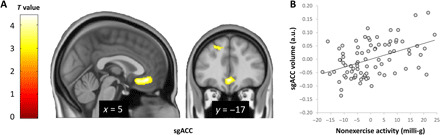
The total amount of nonexercise activity within the study week relates to gray matter volume of the subgenual ACC. (**A**) *T* map of the significant association between mean nonexercise activity across the study week and gray matter volume. For illustration purposes, findings are shown at a significance threshold of *P* < 0.005, uncorrected. (**B**) Scatterplot of the association between mean nonexercise activity across the study week [*x* axis; depicted are group-centered Movement Acceleration Intensity values (for details, see Methods), residualized for age, gender, total intracranial volume (TIV), BMI, and total exercise activity across the study week) and subgenual ACC gray matter volume [*y* axis; depicted are individual gray matter volume values for the peak voxel as defined by the main ROI analysis as shown in (A), residualized for age, gender, TIV, BMI, and total exercise activity across the study week]. a.u., arbitrary units.

As hypothesized (hypothesis I), the region-of-interest (ROI) analysis within our a priori ROI (see Methods) revealed that the total amount of nonexercise activity (parameterized as Movement Acceleration Intensity measured via the accelerometers; for details, see Methods) of participants within the study week was significantly associated with the gray matter volume of the subgenual part of the ACC (sgACC) [*T*(76) = 4.43; *P*_FWE_ = 0.046, corrected within ROI, peak voxel: 0, 36, −10]. Participants with higher nonexercise activity levels showed larger sgACC volume compared with participants who were less physically active in their everyday life (see Fig. 1). No other brain areas outside the a priori ROI reached whole-brain significance. Control analyses showed that these results are specific for nonexercise activities: Mapping the total physical activity intensity (regardless of any distinction) led to a null finding, i.e., there was no significant association of total physical activity intensity with brain gray matter volume within our ROI, therewith strengthening the case for a specific role of nonexercise activity.

#### sgACC volume as a moderator of the within-subject association between nonexercise activity and energetic arousal

To test whether sgACC gray matter volume, which showed a significant association with nonexercise activity, moderated the within-subject association between nonexercise activity and energetic arousal, we applied a multilevel analysis. We nested the energetic arousal assessments within participants. Nonexercise activity (parameterized as Movement Acceleration Intensity measured via the accelerometers; for details, see Methods) within the 60-min segments before each e-diary prompt and individual gray matter volume values extracted from the peak-voxel within the sgACC were predictors of interest. We modeled a cross-level interaction introducing sgACC gray matter volume as a level 2 moderator of the association between nonexercise activity and energetic arousal reported on a validated two-item short scale (range, 0 to 100) on smartphone diaries constituting the dependent variable (see Methods).

**Fig. 2:**
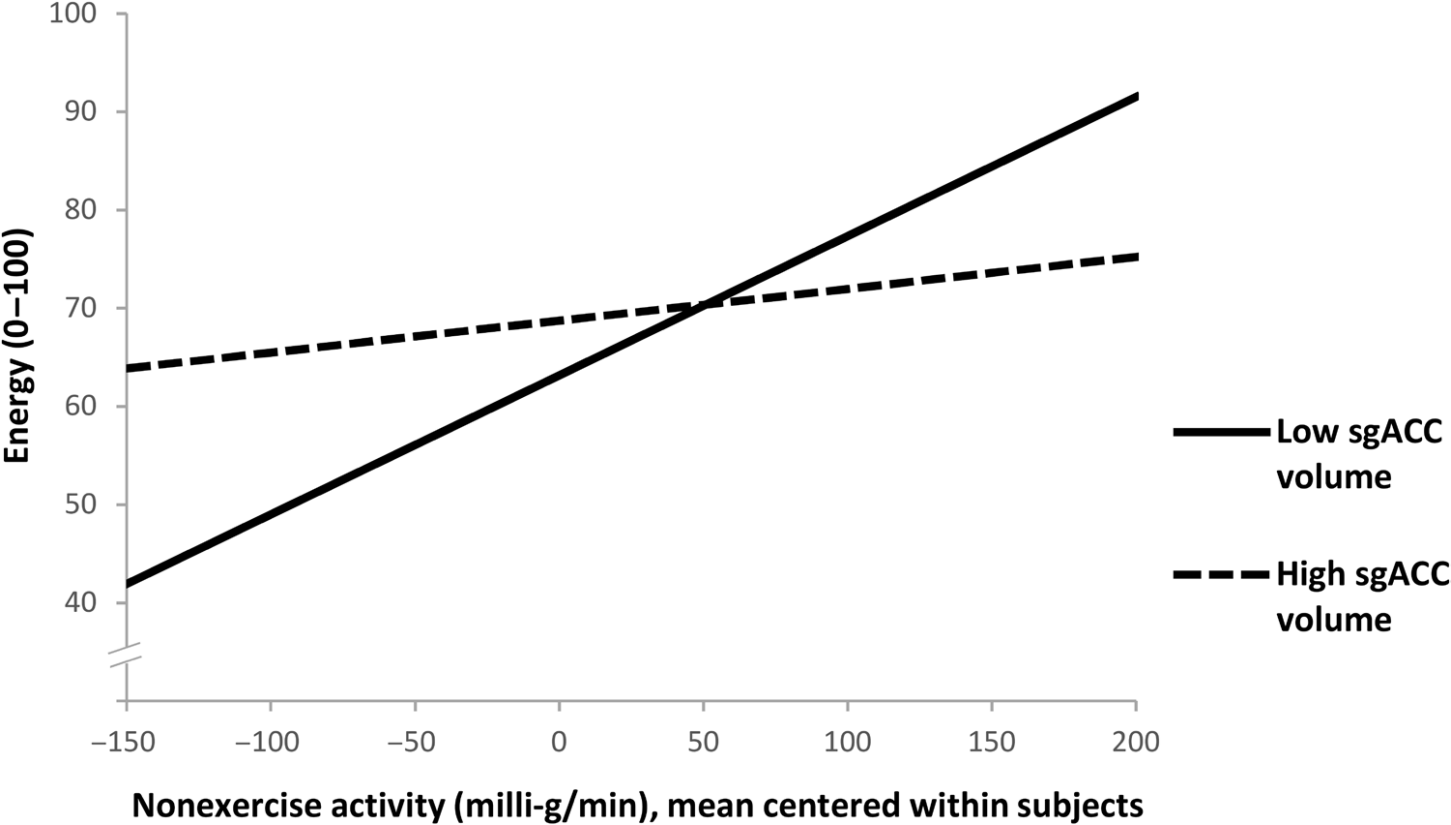
sgACC volume moderates the within-subject association between nonexercise activity and energetic arousal. Here, the estimated within-person effects of nonexercise activity (*x* axis) on energy (*y* axis) within the study participant showing the lowest (solid regression line) versus within the participant demonstrating the highest sgACC volume (dashed regression line) from the multilevel model are displayed. Please note that nonexercise values to the left of the *x* axis refer to sedentary behavior such as sitting, while nonexercise values to the right depict moderate activities such as walking in everyday life.

As hypothesized (hypothesis II), we found a significant interaction effect (*P* = 0.001; see Table 2) revealing that participants with low sgACC volume felt less energized after inactivity, but more energetic after nonexercise activity compared with their counterparts with high sgACC volume (see Fig. 2). Control analyses showed that these results are robust against several potential confounders, such as the socioeconomic status of participants or daily sleep (see table S5 and section S2). While all participants were included in our initial analyses, regardless of their exercise duration, a control analysis showed that the results were robust against the exclusion of potential outliers in exercise duration (see section S3 and figs. S2 and S3).

**Table 2:** Multilevel moderation analysis in the replication study (*n* = 83): sgACC volume moderates within-subject association of nonexercise activity and energetic arousal. Outcome: energetic arousal (range, 0 to 100); presented are the fixed effects. a.u., arbitrary units.

**Predictor**	**β coefficient**	**SE**	***T* value (*df*)**	***P* value**
Intercept	22.578	19.397	1.16 (78.8)	0.248
**Moderation (cross-level interaction analysis)**				
sgACC volume*Nonexercise activity (a.u.)	−0.148	0.046	−3.23 (5532)	0.001
**Main predictors**				
sgACC volume (a.u.)	−8.664	17.799	−0.49 (74.9)	0.628
Nonexercise activity (milli-g)	0.185	0.038	4.92 (5532)	<0.001
**Covariates on level 1 (within-subject)**				
Time (hours)	7.902	0.437	18.09 (5540)	<0.001
Time squared (hours^2^)	−0.279	0.014	−19.43 (5540)	<0.001
Exercise activity/day (min)	−0.020	0.012	−1.69 (5535)	0.090
**Covariates on level 2 (between-subject)**				
Age (years)	−0.590	0.454	−1.30 (75.2)	0.197
Gender: female	0.102	3.022	0.03 (74.2)	0.973
BMI (kg/m^2^)	0.315	0.378	0.83 (75.1)	0.407
Exercise/week (min)	−0.006	0.006	−0.99 (76)	0.327
Neuroticism (0–4)	−6.219	1.586	−3.92 (74.6)	<0.001
Total intracranial volume (a.u.)	0.005	0.015	0.32 (74.5)	0.750

#### Between-subject associations of energetic arousal with affective well-being and mental health metrics

To explore the relevance of our findings from the within-subject level for affective well-being and mental health, we tested for between-subject differences of our within-subject target construct energy. We conducted multivariate regression analyses with mean energetic arousal as captured by the e-diaries and averaged within participants across the study week, predicting a set of established affective well-being and mental health indicators [i.e., World Health Organization (WHO) Well-Being Index (WBI) (*23*), trait anxiety (*24*), sense of coherence (*25*), satisfaction with life (*26*), optimism (*27*), and self-efficacy (*28*)] in a sample comprising all participants from the replication study who provided these data (*n* = 80). Our analyses, which we controlled for age, sex, and socioeconomic status (*29*), showed both robust multivariate [*F*_6,69_ = 5.134; *P* (Wilks’ lambda) < 0.001] and significant univariate associations (see Table 3) of energetic arousal with these mental health metrics. In particular, participants with higher mean energy across the study week reported higher well-being, sense of coherence, satisfaction with life, more optimism, and self-efficacy yet reduced anxiety (see Table 3), thus confirming hypothesis III and therewith showing the relevance of our findings for affective well-being and mental health.

**Table 3:** Univariate associations of energetic arousal with affective well-being and mental health metrics (*n* = 80). Note: Age, gender, and socioeconomic status (*30*) were entered as covariates.

	**β coefficient**	***F*(df1,df2)**	***P* value**
WHO WBI (WBI25)	0.197	19.514(1,74)	<0.001
Trait anxiety (STAI-T)	−0.255	8.278(1,74)	0.005
Sense of coherence (SOC)	0.187	7.188(1,74)	0.009
Satisfaction with life (SWLS)	0.179	11.176(1,74)	0.001
Optimism (LOT-R)	0.33	10.56(1,74)	0.002
Self-efficacy (SWE)	0.129	8.206(1,74)	0.005

## DISCUSSION

Here, we show that specifically nonexercise activity, the most prominent subtype of humans’ physical activities in everyday life, increases feelings of energy within persons. Nonexercise activity over the span of 1 week relates to gray matter volume of a core region implicated in the regulation of affect, risk for, and recovery from mood disorders, namely, the sgACC. Participants engaging in more nonexercise activity showed higher sgACC volume compared with their less physically active counterparts, and this brain structure moderated humans’ momentary energetic arousal gain from nonexercise activity in everyday life. Reduced sgACC volume predicted a stronger susceptibility to everyday life activity, resulting in a stronger adverse impact of physical inactivity on energy, but a concurrently higher gain of energy when being active. In turn, energy across 1 week was positively and robustly related to affective well-being and mental health metrics within our community-based sample. Given its prior evidence of high importance for mental health (*15*), one might be tempted to speculate that our findings not only are relevant for promoting affective well-being in the general population but also could potentially advance mental health prevention and intervention, if replicated in patient samples.

We provide evidence for a relationship between nonexercise activity and gray matter of a brain structure known to be a key regulatory site for emotional processing implicated in psychiatric risk and resilience (*20*, *30*). The ACC is a critical neural hub of brain circuits involved in the regulation of motivation, emotion, and stress response and closely coupled to the limbic system and the hypothalamic-pituitary-adrenal axis (*20*, *30*). Our direct measurements of the neural effects of nonexercise activity agree with previous indirect evidence linking nonexercise-related physical activity to the ACC: the number of blocks walked over 1 week predicted ACC volume at a 9-year follow-up (*31*), and a brisk walking intervention over 6 months increased ACC volume (*32*).

The subregion of the ACC identified in this study has important implications for affective well-being. Whereas the mid and dorsal ACC are known to play a key role in social categorization processes, the sgACC is crucially involved in emotional and motivational processing of social stimuli and has close reciprocal connections to the amygdala, ventral striatum, and the orbitofrontal cortex. As the sgACC also has strong links to the hypothalamus (*33*), the observed region lies at the intersection of circuits implicated in the processing of affect and energetic control that underlie increased cross-system reactivity observed by Merikangas and colleagues (*15*). Because our finding is correlative, our experiment does not show whether high sgACC volume induced more movement (for example, through its role in emotionally motivated behavior) or nonexercise activity induces volume increases, for example, through mechanisms similar to those discussed for physical exercise activity (*17*).

Our findings shed light on the role of nonexercise activity in the regulation of affective well-being in everyday life, i.e., low-sgACC individuals feel worse (i.e., less energized) if they move less. Because reduced brain volume in the sgACC has been repeatedly found in bipolar disorder and major depression (*34*, *35*), and depressive episodes are characterized by reduced physical activity and reduced feelings of energy (*12*–*15*), we speculate that our results could also point toward a potential risk mechanism for mood disorders: Because lower sgACC volume predicted a stronger adverse impact of sedentary behavior on energy, reduced activity levels that sink further during manifest episodes of depression could trigger stronger reductions in perceived energy, which then would reduce activity even further, a vicious circle. Because our assessment did include neither clinical samples nor clinical assessments of mood disorders, this hypothesis remains to be tested in future research in clinical populations.

As our data show that people with low sgACC volume also gain more energetic benefit when achieving high nonexercise activity, this may suggest that targeting nonexercise activity may be especially beneficial for individuals poorly served by existing treatments: Beyond disease status and risk, sgACC volume has been highlighted as a biomarker for mood disorders, and specifically, reduced sgACC volume has been associated with poorer response to conventional treatments including not only antidepressants and psychotherapy but also brain stimulation and electroconvulsive therapy (*36*). Provided that our findings can be substantiated in future (interventional) studies within patient populations, this would further strengthen the case for complementing current mental health prevention and treatment programs with interventions aimed at improving nonexercise activity.

Our findings reveal previously unidentified facets of the interaction of nonexercise activity and affective well-being in everyday life that appear to be especially promising because they are potentially modifiable through simple targeted interventions across the general population. Given that nonexercise activities constitute the predominant part of humans’ everyday life physical activities and affect all humans even if they do not engage in exercise activities, targeting nonexercise activity to maintain and improve affective well-being appears to be a straightforward procedure. Increasing humans’ nonexercise activity by interventions such as using the stairs instead of the elevator and riding a bike instead of a car may be easier to implement in everyday life compared to structured, exhausting, and time-consuming exercise sessions.

There are several questions resulting from our work that merit further discussion. First, we conducted an observational study in real life. This study design comes with high ecological validity and thus enabled us to observe how individuals’ nonexercise activity and energy unfolded in their everyday life. However, observational study designs do not allow for experimental control of contextual influences, nor is it possible to assess all potentially relevant contextual information. Thus, our study did not assess specific reasons for nonexercise activity engagement (e.g., fetching papers from the basement at work), and potential hidden third variables remain elusive. In future work, ecological momentary interventions that enable the integration of experimental manipulation in everyday life offer a possibility to progress toward ecological validity and assessment of causality. This is a critical next step in the research field of physical activity and brain health. Second, and related to the observational character of our study, our data demonstrate a link between sgACC volume and nonexercise activity over the course of 1 week but do not prove that changes in nonexercise activity cause changes in brain volume. Although longitudinal and interventional studies on effects of nonexercise activity on gray matter volume are rare, findings suggest that even modest increases in physical activity may lead to increased gray matter (*31*, *37*). However, in the context of our study, it could also be that stronger feelings of energy after nonexercise activity led to high sgACC subjects moving more, so further interventional studies are needed to address whether changes in nonexercise activity causally affect region-specific brain volumes. If nonexercise activity does cause volume changes, the cellular mechanisms of this systems-level observation also need to be studied. One exciting possibility that needs to be further evaluated involves an increase in neurogenesis by nonexercise activity. Adult neurogenesis of calretinin-positive interneurons was demonstrated in deep layers of the ACC in rats (*38*). Third, again related to the limitations of observational study designs, we aimed to identify neuronal moderators of the best established time-dynamic relationship of nonexercise activity and feelings of energy. While we parameterized energetic arousal as a consequence of nonexercise activity, taking into account the time order, which constitutes one important aspect of causality (*39*), the issue whether nonexercise activity does causally affect feelings of energy has to be resolved, for example, applying ecological momentary interventions to substantiate all causal criteria. Fourth, participants could not wear the accelerometers throughout all types of exercises due to practical issues. The devices were not waterproof; thus, wearing them while swimming was impossible, and participants were instructed to take the accelerometers off during budding-hugging sports because such types of activities may pose harm to the devices. Therefore, to control for exercise activity, we used the most robust exercise parameter measured, i.e., exercise duration being assessed independently of whether participants wore the accelerometers during exercise or not. To test whether exercise intensity may have influenced our results, we conducted additional analyses. However, adding exercise intensity as a covariate did not change our findings (see section S4). Fifth, we investigated neuronal structures underlying a psychological real-life mechanism seen in the general population using a sample of healthy adults without current diagnoses of any severe mental disorder and without including clinical ratings in our analyses. Therefore, our findings need to be validated in studies in clinical populations to substantiate their translational value for interventions in psychiatric disorders.

In summary, we identified a neural correlate underlying the real-life effects of nonexercise activity on energetic arousal in the general population. Our findings reveal that subjects with low sgACC volume exhibit a stronger negative impact of reduced physical activity on feeling energized. However, we also found that the same biomarker (low sgACC volume) predicted increased emotional gain if high nonexercise activity is achieved. These findings give important insights into how the within-subject interaction of physical activity and affective well-being in everyday life is linked to neural correlates in the general population and can thus guide further research on how to intervene on nonexercise activity to improve affective well-being. Therefore, future interventional studies should investigate causal impacts of nonexercise activity on human gray matter volume, and clinical studies should validate the psychological real-life mechanism of nonexercise activity enhancing energetic arousal in patient populations. If these future proposals are able to endorse our findings to patient groups and do show causality of findings, improving nonexercise activity may complement existing prevention and treatment programs as an easily accessible target.

## METHODS

### Study participants and procedures

Participants were randomly drawn from population registers and selected according to a two-stage proportionally layered procedure (stratified by age, sex, and nationality) at the Central Institute of Mental Health (CIMH; Mannheim, Germany). For the current study, the psychiatric-epidemiological center (PEZ) at CIMH recruited 127 (discovery study) and 126 (replication study) healthy adults aged 18 to 28 years who carried study smartphones and accelerometers across 7 days and provided information on their exercise habits. Participants of the replication study additionally underwent structural magnetic resonance imaging (MRI) scans. For ethical and practical reasons (scheduling of MRI scanning times), we informed the participants before the study started and before the ambulatory assessment study week about the MRI measurement afterward. Participants with chronic endocrine, cardiovascular, immunological, or acute diseases or moderate to severe impairment of intelligence, and participants unable to legally consent were excluded. In addition, participants were excluded if they reported standard MRI contraindications (e.g., metal implants and pregnancy). Following established procedures, we excluded participants if the following criteria applied: (i) severe technical problems with the accelerometer such as a prematurely terminated measurement (discovery study: *n* = 13; replication study: *n* = 10) and (ii) e-diary compliance below 30% (discovery study: *n* = 1; replication study: *n* = 1). In addition, to ensure utmost data quality, we excluded participants if any insufficient wear time of accelerometers following established procedures (*40*) and any implausible exercise reports (see details below) were detected (discovery study: *n* = 46; replication study: *n* = 32). The final discovery sample consisted of 67 healthy participants (49 females) with a mean age of 23.4 years (SD = 2.9; see table S3), and the final replication sample consisted of 83 healthy participants (42 females) with a mean age of 23.3 years (SD = 2.6; Table 1). All participants provided written informed consent and received monetary compensation for study participation. After an extensive technical briefing, including individual testing at the PEZ, participants carried a study smartphone and an accelerometer (movisens Move II/III) for seven consecutive days. Thereafter, participants returned the devices, reported on their exercise activities, and participated in a structural MRI scan (only replication study). To optimize participants’ recall when reporting on exercise activities, we applied an established procedure similar to the Day Reconstruction Method (*41*); in short, participants were shown all locations visited and routes covered (tracked via smartphone) on a time-stamped digital map (software movisens Geocoder) and were asked to label their exercise activities (i.e., point in time, exercise type, and duration). To ensure utmost data quality, we (i) plotted accelerometer data [software FZI (Forschungszentrum Informatik) and movisens UnisensViewer] and checked whether the reported exercise time points were face valid (i.e., increased acceleration signal within the reported exercise time frame or zero acceleration signal, e.g., if participants engaged in swimming and thus did not wear the accelerometer). In addition, we (ii) displayed the location profiles of all participants on a digital map (software movisens Geocoder) and identified implausible exercise locations by visual inspection. For any identified errors in exercise reports, participants were recontacted. If implausibility could not be fixed, participants were excluded from the data analyses (see above).

The study was approved by the medical ethics committee II of the Medical Faculty Mannheim at the Ruprecht Karls University in Heidelberg and fulfilled the ethical guidelines for medical research according to the Declaration of Helsinki. Written and oral information regarding study procedures were presented to all eligible participants before written informed consent was obtained. There was no surrogate consent procedure. All participants were free to withdraw from the study at any time.

### Data assessment and preprocessing

#### Psychological data assessment

Before the ambulatory assessment procedure, participants underwent psychological data assessment. They completed a battery of questionnaires and self-ratings including basic sociodemographic information, an established multidimensional index of socioeconomic status based on retrospective self-ratings of occupational status, educational attainment, and household income (SES21) developed by Lampert *et al.* (*29*); a retrospective self-rating inventory to quantify trait neuroticism (NEO Five-Factor Inventory–30) (*42*); a retrospective five-item self-rating of subjective well-being across the prior 2 weeks (WHO WBI, WBI25) (*23*); and self-rated subjective social status (MacArthur Scale of Subjective Social Status) (*43*). Moreover, participants completed a 20-item self-rating of trait anxiety [State-Trait Anxiety Inventory (STAI-T) (*24*)], a 29-item self-rating inventory capturing sense of coherence [Sense of Coherence Scale (*25*)], a 5-item self-rating scale assessing satisfaction with life [Satisfaction With Life Scale (SWLS) (*26*)], a 3-item subscale optimism of the self-reported Life Orientation Test [LOT-R; (*27*)], and a 10-item self-rating of self-efficacy [SWE; (*28*)]. Participants’ body mass index (BMI) was calculated from self-reported data on height and weight.

#### Nonexercise activity

Participants wore accelerometers (right hip) during the entire study week but not during sleep. The triaxial acceleration sensors captured movements of as much as ±8 g with a sampling frequency of 64 Hz and a resolution of 12 bits and were shown to be appropriate in assessing human physical activity (*44*). To compute Movement Acceleration Intensity for quantification of nonexercise activity, i.e., the vector magnitude of the acceleration in milli-g [(g)/1000] assessed at the three sensor axes, we used the software movisens DataAnalyzer (version 1.6.12129). In short, gravitational components were eliminated by a high-pass filter (0.25 Hz), and artifacts (e.g., vibrations when cycling on a rough road surface or shocks of the sensor) were eliminated by a low-pass filter (11 Hz). To test whether nonexercise activity relates to the sgACC volume, we aggregated the minutely Movement Acceleration Intensity values across the whole study week for each individual and subjected this parameter as a level 2 predictor of interest into our MRI statistics (see below). To merge the accelerometer and the e-diary data, we used the software movisens DataMerger (version 1.6.3868). To focus our analyses on nonexercise activity, we excluded all Movement Acceleration Intensity data within the time frames where participants had been exercising.

For within-subject analyses, we followed established procedures and averaged Movement Acceleration Intensity across 60-min segments prior to each e-diary assessment as those intervals of physical activity were shown to be highly correlated with both acute energy ratings and daily physical activity levels and because there is evidence for transdiagnostic cross-system reactivity for those physical activity bouts (*15*). This parameter, a continuous variable with a natural zero, entered our multilevel models as level 1 predictor of interest (see below).

#### Ecological momentary assessment of energetic arousal

Traditional e-diary sampling strategies (e.g., time-based querying for mood every hour) have been shown to be inferior compared to interactive trigger algorithms, as fixed assessments such as every hour may, by chance, not be linked to any high physical activities, thus limiting the conclusiveness of the data. Therefore, we implemented a mixed sampling strategy including a custom-developed GPS-based trigger algorithm on the smartphones using the experience sampling software movisensXS (xs.movisens.com; version 0.6.3658). In particular, the GPS-based algorithm triggered energetic arousal assessments every time participants covered distances larger than 500 m. However, assessments were triggered not more often than every 40 min but at least every 100 min even if participants had not moved. In addition, triggers at fixed times (at 08:00 and 22:20) were implemented. The smartphone prompted the participants via an acoustic, visual, and vibration signal and offered the chance to postpone a prompt up to 15 min.

We used a two-item short scale to assess energetic arousal, which is based on the originally German language Multidimensional Mood Questionnaire and comprised the bipolar items (German translations) “without energy”-“full of energy” (“energielos”-“energiegeladen”) and “tired-awake” (“müde”-“wach”) presented in a mixed order and reversed polarity on visual analog scales (range, 0 to 100). The scale demonstrated suitable psychometric properties, i.e., reliability coefficients of 0.90 on the between-person level and 0.77 on the within-person level, showing that this scale is suitable to assess fluctuations of energetic arousal on the within-person level over time on e-diaries. The mean of the item scores entered our multilevel analyses as the dependent variable.

#### Magnetic resonance imaging

MRI was acquired on a 3-T whole-body Siemens Magnetom Trio scanner at the CIMH using a T1-weighted three-dimensional magnetization-prepared rapid acquisition gradient-echo (MPRAGE) sequence. Structural scans were acquired with whole-brain coverage, a spatial resolution of 1 mm^3^, and the following specifications: repetition time = 2300 ms, echo time = 3.03 ms, inversion time = 900 ms, flip angle = 9°, 192 contiguous sagittal slices, 1-mm slice thickness, field of view = 256 mm. Voxel-based morphometry (VBM) is an automated whole-brain processing method implemented in the Computational Anatomy Toolbox (CAT; www.neuro.uni-jena.de/cat/index.html#VBM), which allows to segment the composition of brain tissue into gray matter, white matter, and cerebral spinal fluid (*45*). Image processing followed standard procedures, including tissue classification and segmentation into gray matter, white matter, cerebrospinal fluid, and noncerebral tissue classes. Normalization to Montreal Neurological Institute (MNI) space was done with a diffeomorphic image registration algorithm (DARTEL). In addition, correction for image intensity nonuniformity, cleaning up of gray matter partitions, the application of a hidden Markov random field model, and spatial adaptive nonlocal means denoising were applied. The resulting tissue segments were multiplied by the Jacobian determinants of the deformation field to transform the gray matter density values into volume equivalents. Total intracranial volume was computed for each participant and added as a covariate to group analyses. The segmented, normalized, noise-corrected, and modulated gray matter images were then smoothed with an 8-mm full width at half maximum isotropic Gaussian kernel. VBM analysis was conducted following the standard procedures using the CAT implemented in the Statistical Parametric Mapping (SPM) software (SPM12) executed in MATLAB R2013b. Raw images were visually inspected for scanner artifacts and structural abnormalities.

### Statistical analyses

#### MRI data analysis

Individual preprocessed gray matter volume maps were analyzed in SPM12 using a mass univariate general linear model. To test whether the total amount of nonexercise activity within the study week related to the gray matter volume, we computed a general linear model with nonexercise activity as regressor of interest, and age, gender, total intracranial volume, the total amount of exercise activities within the study week, and BMI as covariates. On the basis of previous findings (*16*–*21*, *46*), we conducted an ROI analysis in an a priori defined mask (16,127 voxels) comprising anterior cingulate [Brodmann areas (BA32 and BA24)], prefrontal (lateral BA9 and BA46), premotor (lateral BA6) cortices, amygdala, thalamus, and hippocampus [based on Automated Anatomical Labeling in SPM12 (*47*)]. For the ROI analysis, we used the small volume correction approach implemented in SPM12. The significance threshold for the ROI analysis was set to *P* < 0.05 family-wise error (FWE) corrected for multiple comparisons across all voxels within the mask. Outside this prehypothesized ROI, findings were considered significant if they passed a significance threshold of *P* < 0.05 FWE corrected for multiple comparisons across the whole brain. For each participant, we extracted individual gray matter values for moderation analyses from the peak voxel in the sgACC as defined by the main ROI analysis.

#### Multilevel models for within-subject (moderation) analyses

In our discovery study, to test whether the association of physical activity and energetic arousal is indeed driven by nonexercise activities, we applied a multilevel analysis nesting energetic arousal assessments (level 1) within participants (level 2). We successively entered the predictors of interest, i.e., nonexercise activity within the 60-min segments before each e-diary prompt and the cumulative exercise activity duration per day (min) (both level 1) into our model. We entered the level 1 covariates previously shown to influence within-subject fluctuations of energetic arousal, i.e., time of the day (hours) and time of the day squared (hours^2^). Thereafter, we entered level 2 covariates, i.e., age (years), gender, BMI (kg/m^2^), and total exercise duration per week (min).

In Eq. 1, the multilevel model is detailed. Here, on level 1, within-subject effects were estimated with participants’ e-diary entries (subscript *j*) at any time of measurement (subscript *i*). *Y_ij_* represents the level of energetic arousal in person *j* at the time *i.* β coefficients represent the intercept and the effects of nonexercise activity, cumulative exercise activity duration per day, time of the day, time of the day squared, at level 1, and *r_ij_* represents the residuals at level 1. We centered the predictors time and time squared, subtracting the start time of the study for each day (7:30) and centered nonexercise activity as well as cumulative exercise activity duration per day around the participants’ mean. On level 2, between-subject effects were estimated (predictors: age, gender, BMI, neuroticism, and total exercise duration per week). We included random effects for the intercept (*u*_0*j*_).

Equation 1

$$\begin{array}{c} Y{\left(\text{energetic arousal}\right)}_{\mathrm{ij}}\\ =\beta_{00}+\beta_{01}*{\text{age}}_{j}+\beta_{02}*{\text{gender}}_{j}+\beta_{03}*{\text{BMI}}_{j}+\beta_{04}*{\text{neuroticism}}_{j}\\ \;\;+\beta_{05}*\text{total exercise duration per}\;{\text{week}}_{j}\\ \;\;+\beta_{10}*\text{non}\;{\text{exercise activity}}_{\text{ij}}\\ \;\;+\beta_{20}*\text{cumulative exercise activity duration per}\;{\text{day}}_{\mathrm{ij}}\\ \;\;+\beta_{30}*{\text{time of day}}_{ij}+\beta_{40}*{\text{time of day}}_{\mathrm{ij}}^{2}+u_{0j}+r_{\mathrm{ij}} \end{array}$$

In our replication study, to test whether sgACC gray matter volume (which showed a significant association with nonexercise activity in the main analysis) moderated the within-subject association between nonexercise activity and energetic arousal, we again applied exactly the same multilevel analysis as for our discovery study. However, we additionally entered the predictor peak voxel gray matter of the sgACC (level 2) into our model. Moreover, we additionally entered total intracranial volume [arbitrary units (a.u.)] as a level 2 predictor. Last, we modeled a cross-level interaction introducing sgACC gray matter volume as a level 2 moderator of the association between nonexercise activity and energetic arousal.

In Eq. 2, the multilevel model to test whether sgACC volume moderated within-subject associations between nonexercise activity and energetic arousal is detailed. Again, on level 1, within-subject effects were estimated with the participants’ e-diary entries (subscript *j*) at any time of measurement (subscript *i*). *Y_ij_* represents the level of energetic arousal in person *j* at time *i.* β coefficients represent the intercept and the effects of nonexercise activity, time of the day, time of the day squared, cumulative exercise activity duration per day, at level 1, and *r_ij_* represents the residuals at level 1. Again, we centered the predictors time and time squared, subtracting the start time of the study for each day (7:30) and centered nonexercise activity as well as cumulative exercise activity duration per day around the subjects’ mean. On level 2, between-subject effects were estimated (predictors: sgACC volume, age, gender, BMI, neuroticism, total exercise duration per week, and total intracranial volume). We included random effects for the intercept (*u*_0*j*_). For all multilevel analyses, we used the software SAS (SAS 9.4, SAS Institute Inc., Cary, NC, USA) and set the α level to 0.05.

Equation 2

$$\begin{array}{c} Y{\left(\text{energetic arousal}\right)}_{\mathrm{ij}}\\ =\beta_{00}+\beta_{01}*{\text{sACC volume}}_{j}+\beta_{02}*{\text{age}}_{j}+\beta_{03}*{\text{gender}}_{j}+\beta_{04}*{\text{BMI}}_{j}\\ \;\;+\beta_{05}*{\text{neuroticism}}_{j}+\beta_{06}*\text{total exercise duration per}\;{\text{week}}_{j}\\ \;\;+\beta_{07}*{\text{total intracranial volume}}_{j}+\beta_{10}*\text{non}{\text{exercise activity}}_{\mathrm{ij}}\\ \;\;+\beta_{20}*{\text{time of day}}_{\mathrm{ij}}+\beta_{30}*{\text{time of day}}_{\mathrm{ij}}^{2}\\ \;\;+\beta_{40}*\text{cumulative exercise activity duration per}\;{\text{day}}_{\mathrm{ij}}\\ \;\;+\beta_{11}*{\text{s}g\text{ACC volume}}_{j}*\text{non}{\text{exercise activity}}_{\mathrm{ij}}+u_{0j}+r_{\mathrm{ij}} \end{array}$$

Please note that a significant within-subject association between nonexercise activity and energetic arousal has already been published for a subsample of the replication study (overlap with the current analyses: *n* = 38 participants; 45.8%). Thus, a significant main effect of nonexercise activity on energetic arousal was expected for the current replication study, too.

#### Multivariate regression model for cross-validation: Between-subject associations of mean energy with affective well-being and mental health metrics

To explore the relevance for affective well-being and mental health, we tested for associations of the within-subject target energy with established well-being and mental health metrics. In particular, we applied a multivariate regression model followed by univariate analyses and entered mean energetic arousal as captured by the e-diaries and averaged within participants across the study week as predictor, the respective scores of established affective well-being and mental health indicators [i.e., WHO WBI (*23*), trait anxiety (*24*), sense of coherence (*25*), satisfaction with life (*26*), optimism (*27*), and self-efficacy (*28*)] as outcome variables, as well as age, sex, and socioeconomic status (*29*) as covariates into the model. We applied the Wilks’ lambda test statistic for the multivariate test and performed the analyses with the software SPSS (IBM, version 26.0.0.0).

## Supplementary Material

http://advances.sciencemag.org/cgi/content/full/6/45/eaaz8934/DC1

Adobe PDF - aaz8934_SM.pdf

A neural mechanism for affective well-being: Subgenual cingulate cortex mediates real-life effects of nonexercise activity on energy

## SUPPLEMENTARY MATERIALS

Supplementary material for this article is available at http://advances.sciencemag.org/cgi/content/full/6/45/eaaz8934/DC1

## Appendix

View/request a protocol for this paper from *Bio-protocol*.
