# Supplementary material for: A neural mechanism for affective well-being: Subgenual cingulate cortex mediates real-life effects of nonexercise activity on energy
Source: Sci Adv. 2020 Nov 6;6(45):eaaz8934. doi: 10.1126/sciadv.aaz8934 (PMC7673710; doi:10.1126/sciadv.aaz8934)
Supplement: http://advances.sciencemag.org/cgi/content/full/6/45/eaaz8934/DC1 [file supp_6_45_eaaz8934__index.html]

Science Advances | Science AdvancesAAASSearchScience AdvancesMenu

## Supplementary Materials

# A neural mechanism for affective well-being: Subgenual cingulate cortex mediates real-life effects of nonexercise activity on energy

Markus Reichert, Urs Braun, Gabriela Gan, Iris Reinhard, Marco Giurgiu, Ren Ma, Zhenxiang Zang, Oliver Hennig, Elena D. Koch, Lena Wieland, Janina Schweiger, Dragos Inta, Andreas Hoell, Ceren Akdeniz, Alexander Zipf, Ulrich W. Ebner-Priemer, Heike Tost, Andreas Meyer-Lindenberg

Download Supplement

**This PDF file includes:**

- Figs. S1 to S3
- Sections S1 to S4
- Tables S1 to S5

**Files in this Data Supplement:**

- Adobe PDF - aaz8934\_SM.pdf
