# Supplementary material for: A neural mechanism for affective well-being: Subgenual cingulate cortex mediates real-life effects of nonexercise activity on energy
Source: Sci Adv. 2020 Nov 6;6(45):eaaz8934. doi: 10.1126/sciadv.aaz8934 (PMC7673710; doi:10.1126/sciadv.aaz8934)
Supplement: http://advances.sciencemag.org/cgi/content/full/6/45/eaaz8934/DC1 [file supp_6_45_eaaz8934__1.pdf]

Science Advances
AAAS

advances.sciencemag.org/cgi/content/full/6/45/eaaz8934/DC1

Supplementary Materials for

A neural mechanism for affective well-being: Subgenual cingulate cortex mediates real-life effects of nonexercise activity on energy

Markus Reichert*, Urs Braun, Gabriela Gan, Iris Reinhard, Marco Giurgiu, Ren Ma, Zhenxiang Zang, Oliver Hennig, Elena D. Koch, Lena Wieland, Janina Schweiger, Dragos Inta, Andreas Hoell, Ceren Akdeniz, Alexander Zipf, Ulrich W. Ebner-Priemer, Heike Tost, Andreas Meyer-Lindenberg

*Corresponding author. Email: markus.reichert@zi-mannheim.de

Published 6 November 2020, *Sci. Adv.* **6**, eaaz8934 (2020)
DOI: 10.1126/sciadv.aaz8934

This PDF file includes:

Figs. S1 to S3
Sections S1 to S4
Tables S1 to S5

Figure S1. Research plan

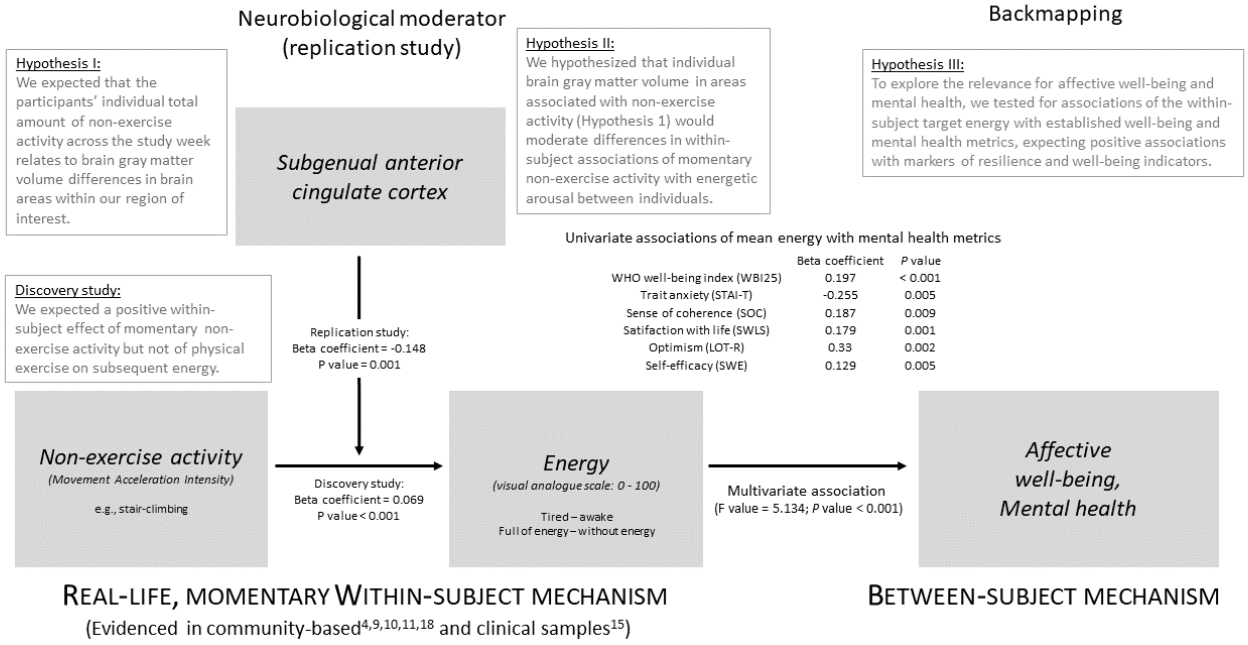

Section S1. Robustness against alternative definitions of physical activities

To test if the definition of physical activities used may have influenced our results, we conducted additional analyses. While the definition of exercise vs. non-exercise activities considers both physiological and psychological processes involved in physical activity (e.g., the (social) context, motive, structure, duration, and energy expenditure; for details see introduction section and (*4*)), a more parsimonious definition following categories of physical activities derived from energy expenditure estimates that are well-established and broadly used (e.g., to investigate effects of physical activity on somatic health outcomes), can be vigorous vs. light/moderate physical activity, therewith focusing on the physiological aspects of physical activity.

Therefore, to test if the latter and alternative definition of physical activities influences our results, we conducted a series of additional analyses using the extant analyses procedures but the definition of vigorous vs. light/moderate physical activity instead of the differentiation non-exercise vs. exercise activity. First, participants' physical activity levels were correlated across the different definitions, see Table S1. Second, in our discovery study, using the definition of vigorous vs. light/moderate physical activity instead of the differentiation non-exercise vs. exercise activity lead to the same results, i.e., a significant effect of light/moderate activity yet a non-significant result for vigorous activity on energetic arousal (see Table S2). Third, control analyses in the replication study further substantiated this finding: light/moderate activity but not vigorous activity predicted energetic arousal (see Table S2).

Together, these additional analyses using the alternative definition of physical activities lead to the same findings as the distinction of exercise vs. non-exercise activities therewith providing further evidence that momentary positive effects of physical activity on energetic arousal in real-life are specifically driven by every-day activities of light/moderate intensity (which are most prevalent in human life; https://sites.google.com/site/compendiumofphysicalactivities/) but not by vigorous activities (oftentimes characterizing physical exercise sessions; https://sites.google.com/site/compendiumofphysicalactivities/.

Table S1. Correlations of physical activities across different definitions of activity

**Pearson correlations (person level)**	**Discovery study**	**Replication study**
Mean MAI within light/moderate and non-exercise activity categorization	r = 0.881; P value < 0.001	r = 0.883; P value < 0.001
Mean MAI within vigorous and exercise activity categorization	r = 0.581; P value < 0.001	r = 0.525; P value < 0.001

For details on these analyses, refer to Section S1.

Table S2. Control analyses for robustness against alternative definitions of physical activities

Physical activity category	Discovery study	Replication study
light/moderate physical activity	Beta coefficient = 0.057; *P* value < 0.001	Beta coefficient = 0.061; *P* value < 0.001
vigorous physical activity	Beta coefficient = 0.000; *P* value = 0.321	Beta coefficient = 0.000; *P* value = 0.754

For details on these analyses, refer to Section S1.

Section S2. Robustness against sleep as a potential confounder

To test whether sleep may confound our findings, we conducted additional analyses. We operationalized sleep using the accelerometer data. Since participants had been thoroughly instructed to wear the devices throughout all waketimes but not during sleep, we quantified sleep duration via the non-wear times of the accelerometers. In particular, we parameterized sleep duration as the daily amount of accelerometer non-wear time during night times, which we defined as the time frame from 22.30 to 07.30 since participants received e-diary prompts each day between 7:30 and 22:30. Put simply, this sleep parameter represents the non-wear time between 22:30 and 7:30 for each night. We entered this parameter into our main analysis as an additional within-subject variable on a day level (sleep minutes per participant and per day), which lead to a three-level multilevel model for this additional analysis: measurements (on level 1) were nested within days (on level 2) and participants (on level 3). However, incorporating sleep duration (i.e., controlling for potential effects of sleep duration within the night on the e-diary ratings of the following day) did not alter our findings (Interaction analyses sgACC*non-exercise activity: Beta coefficient = -0.148; *P* value = 0.001).

Section S3. Robustness against potential outliers in exercise duration

We researched if potential outliers in exercise duration, i.e., data of participants engaging in relatively high amounts of exercise across the study week influenced our results. Therefore, we first explored the distribution of exercise duration per participant and study week in the discovery vs. the replication study, see Figures S1 and S2 below. Given the distribution of the data, we decided to define outliers as exercise duration > 600 minutes/participant/week, yielding two potential outliers in the replication study. However, excluding these outliers from our main analysis did not change the results (Interaction analyses: sgACC*non-exercise activity: Beta coefficient = -0.150; *P* value = 0.001).

Figure S2. Distribution of exercise duration in the discovery study

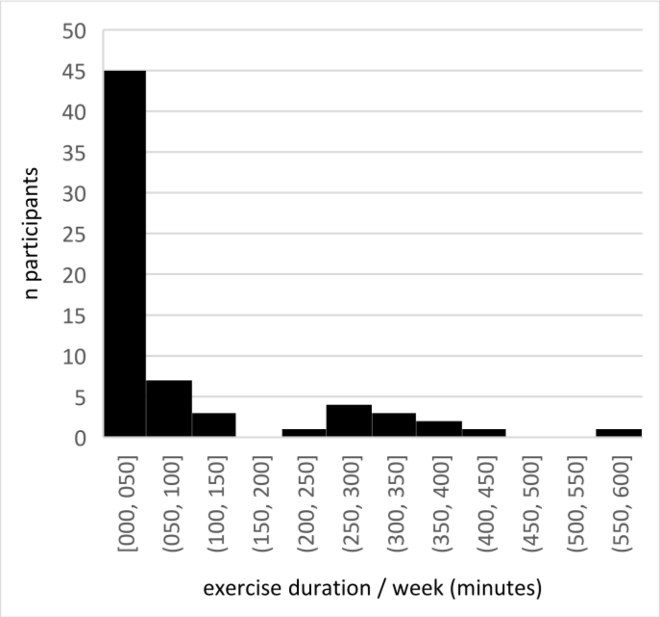

The histogram depicts the distribution of the cumulative exercise duration across the study week (in minutes; x-axis), in which participants of the discovery study (y-axis) engaged in; 43 of a total of 67 participants did not engage in any physical exercise at all across the study week.

Figure S3. Distribution of exercise duration in the replication study

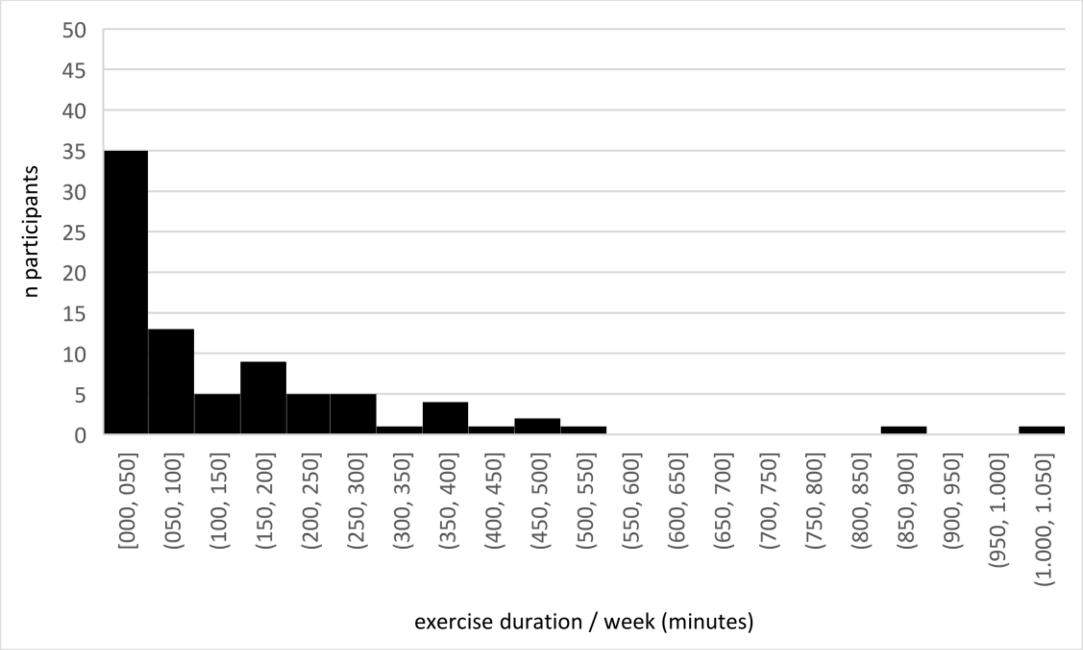

The histogram depicts the distribution of the cumulative exercise duration across the study week (in minutes; x-axis), in which participants of the replication study (y-axis) engaged in; 33 of a total of 83 participants did not engage in any physical exercise at all across the study week.

Section S4. Robustness against exercise intensity as a potential confounder

To test if exercise intensity may have influenced our results, we conducted additional analyses. Therefore, we operationalized exercise intensity in two different ways. First, as the mean Movement Acceleration Intensity (MAI; see methods section for details) across all exercise activities when participants wore the accelerometers. Second, to compensate for missing accelerometer data during exercise sessions when participants could not wear the devices (such as swimming), we manually assigned metabolic equivalent (MET) values to all exercise labels that had been reported by the participants after the study week via an advanced day reconstruction method (see methods section and (*42*)), using the recognized MET compendium by Ainsworth and colleagues (https://sites.google.com/site/compendiumofphysicalactivities/).
However, adding exercise intensity as an additional covariate into our main analysis did not change the results (Interaction analyses sgACC*non-exercise activity using the operationalization of exercise intensity via MAI: Beta coefficient = -0.148; *P* value = 0.001; Interaction analyses sgACC*non-exercise activity using the operationalization of exercise intensity via MET: Beta coefficient = -0.148; *P* value = 0.001).

Table S3. Participant characteristics and descriptives of the discovery study (n = 67)

	min	max	mean	SD
Age [years]	18	28	23.42	2.95
BMI [kg/m²]	17.01	31.51	22.27	3.26
Socioeconomic status (SES)	7.90	20.80	14.62	3.30
World Health Organization Well-being Index (WBI)	8	23	16.34	3.90
Neuroticism (NEO-FFI-30-N)	0.17	3.17	1.32	0.68
Non-exercise activity [milli-g/participant/week]	22.66	86.78	49.28	14.12
Energetic arousal [mean/participant/week]	35.12	85.49	57.41	10.41
Exercise activity [min/participant/week][#]	0	562	73.55	133.49
E-diary compliance (answers/queries in [%])	51.22	100	84.92	12.51

Abbreviations: BMI – Body Mass Index, WBI – Well-Being Index, NEO-FFI-30 – NEO−Five−Factor Inventory (short version). [#]When excluding data of participants who did not at all engage in exercise throughout the study week, there remains no difference between the discovery vs. the replication study with regard to the mean exercise duration of participants that engaged in exercise (mean (discovery study; n = 24) = 205.33 min, SD = 151.34 min; mean (replication study; n = 50) = 229.96 min; t (72) = -0.536; *P* value = 0.594).

Table S4. Multilevel analysis in the discovery study (n = 67): non-exercise activity predicts energetic arousal [range 0-100] within-subjects; presented are the fixed effects

**Predictor**	**Beta coefficient**	**Standard Error**	**T value (df)**	***P* value**
Intercept	66.945	11.891	5.63 (61.3)	< 0.001
**Main predictors (level 1: within-subject)**				
Non-exercise activity [milli-g]	0.069	0.006	11.97 (4846)	< 0.001
Exercise activity/day [min]	0.017	0.013	1.25 (4845)	0.212
**Covariates on level 1 (within-subject)**				
Time [hours]	4.438	0.251	17.70 (4850)	< 0.001
Time-squared [$hours^2$]	-0.323	0.016	-20.14 (4850)	< 0.001
**Covariates on level 2 (between-subject)**				
Age [years]	0.147	0.414	0.35 (61.1)	0.725
Gender: female	1.263	2.838	0.44 (61.3)	0.658
BMI [kg/m^2]	-0.583	0.367	-1.59 (60.8)	0.117
Exercise/week [min]	-0.003	0.009	-0.35 (60.5)	0.726
Neuroticism [0–4]	-7.140	1.790	-3.99 (61.4)	< 0.001

Abbreviations: BMI – Body Mass Index.

Table S5. Robustness against socioeconomic status as a potential confounder

**Covariate**	**Interaction analyses:**<br>**sgACC*non-exercise activity**
Socioeconomic status (as quantified by Lampert et al.'s questionnaire (*30*))	Beta coefficient = -0.148; *P* value = 0.001
Perceived social status (as quantified by the MacAthur scale (*44*))	Beta coefficient = -0.148; *P* value = 0.001
Socioeconomic status (as quantified by Lampert et al.'s questionnaire (*30*))<br>and<br>Perceived social status (as quantified by the MacAthur scale (*44*))	Beta coefficient = -0.148; *P* value = 0.001

To test whether the socioeconomic status of the participants may have confounded our results, we conducted additional analyses, entering socioeconomic status as quantified by Lampert et al.'s questionnaire (*30*), and perceived social status derived from the MacAthur scale ((*44*); see Methods section for details) into our main analysis (either as single predictors or both predictors together). However, adding the additional covariates did not change our findings.
